# Copy Number Heterogeneity in the Virulence Plasmid of *Salmonella enterica*

**DOI:** 10.3389/fmicb.2020.599931

**Published:** 2020-12-04

**Authors:** María A. Sánchez-Romero, Ángela Mérida-Floriano, Josep Casadesús

**Affiliations:** Departamento de Genética, Facultad de Biología, Universidad de Sevilla, Sevilla, Spain

**Keywords:** Salmonella, virulence plasmid, copy number, phenotypic heterogeneity, noise

## Abstract

Quantitative PCR analysis shows that the virulence plasmid of *Salmonella enterica* serovar Typhimurium (pSLT) is a low-copy-number plasmid, with 1–2 copies per chromosome. However, fluorescence microscopy observation of pSLT labeled with a *lacO* fluorescent tag reveals cell-to-cell differences in the number of foci, which ranges from 1 to 8. As each focus must correspond to ≥1 plasmid copy, the number of foci can be expected to indicate the minimal number of pSLT copies per cell. A correlation is found between the number of foci and the bacterial cell volume. In contrast, heterogeneity in the number of foci appears to be independent of the cell volume and may have stochastic origin. As a consequence of copy number heterogeneity, expression of a pSLT-bone reporter gene shows high levels of cell-to-cell variation, especially in actively dividing cultures. These observations support the notion that low-copy-number plasmids can be a source of gene expression noise in bacterial populations.

## Introduction

Plasmid-encoded genes of bacterial pathogens can influence multiple aspects of the pathogen-host interaction, such as adhesion to host surfaces, invasion of host cells, intracellular survival, colonization of organs, and modulation of host immune responses (Pilla and Tang, 2018). In *Salmonella enterica*, plasmids that encode virulence determinants were described several decades ago (Gulig et al., 1993). More recent studies have identified plasmid-borne genes that control the expression of chromosomal loci such as the flagellar network and genes encoding efflux pumps (Huttener et al., 2019; Lian et al., 2019). An unsuspected finding is the existence of *Salmonella* plasmids that harbor essential genes (Canals et al., 2019). The *Salmonella* plasmidome is also a massive reservoir of antimicrobial resistance determinants (Emond-Rheault et al., 2020).

In serovar Typhimurium, most strains carry a plasmid of 50–100 kb known as the *Salmonella* virulence plasmid (Gulig et al., 1993; Guiney et al., 1994; Baumler et al., 1998). This plasmid was designated virulence plasmid of *S. enterica* serovar Typhimurium (pSLT) in strain LT2 (Jones et al., 1982), and the acronym was later extended to sibling plasmids of other *Salmonella Typhimurium* strains (Rotger and Casadesus, 1999).

Plasmid pSLT belongs to the MOB_F1_ group on the basis of relaxase classification (Garcillan-Barcia et al., 2009) and harbors genes for conjugal transfer (*tra*) homologous to those of the F sex factor (Garcia-Quintanilla and Casadesus, 2011); however, *tra* gene deletions are relatively common (Rotger and Casadesus, 1999). All *Salmonella* virulence plasmids share a ~8 kb region, *Salmonella* plasmid virulence (*spv*), required for bacterial proliferation in the phagocytic system of warm-blooded vertebrates (Gulig et al., 1993; Rotger and Casadesus, 1999). Additional pSLT genes involved in pathogenesis are the *pef* fimbrial operon (Baumler et al., 1996), the *rck* gene which confers resistance to complement killing (Heffernan et al., 1992) and promotes epithelial cell invasion (Cirillo et al., 1996), and the *rsk* gene, involved in resistance to host complement (Vandenbosch et al., 1989). Hybrid plasmids harboring virulence and antibiotic resistance determinants have been described in *S. enterica* clinical isolates (Guerra et al., 2002; Mendoza et al., 2009).

Plasmid pSLT has a low (≥1) copy number per cell (Camacho et al., 2005), and possesses at least three maintenance mechanisms: control of replication and copy number mediated by homologs of F plasmid RepB and RepC (Tinge and Curtiss, 1990), a canonical partition system, *parAB* (Tinge and Curtiss, 1990), and two toxin-antitoxin systems, *ccdABST* and *vapBC2_ST_* (Lobato-Marquez et al., 2015). Co-operative activity of such systems may explain the remarkable stability of pSLT, whose frequency of spontaneous curing is around 10^−7^ per cell and generation under laboratory conditions (Garcia-Quintanilla et al., 2006).

In this study, we have used fluorescence microscopy and flow cytometry to track pSLT in individual *S. enterica* cells. We provide evidence that the pSLT copy number is heterogeneous, especially among dividing cells. Tight control of pSLT replication and partition (Lobato-Marquez et al., 2015) is thus compatible with cell-to-cell differences that produce gene expression noise at the population level. We discuss the possibility that copy number heterogeneity might be an adaptive trait.

## Materials and Methods

### Bacterial Strains, Plasmids, and Growth Conditions

*Salmonella enterica* strains used in this study belong to serovar Typhimurium and derive from ATCC 14028 (Table 1). For construction of strain SV8214, a *lacO* cassette consisting of 48 repeats of the lactose operator was integrated into the *tra* operon of the virulence plasmid. In the initial step of pSLT-*lacO* construction, the *lacO* array was PCR-amplified from p48LacO, a derivative of pLAU07 (Lau et al., 2003) obtained from David Sherratt’s laboratory (University of Oxford, England). Amplification was achieved using primers lacO1 and lacO2 (Supplementary Table S1), and the amplified fragment was cloned onto pDOC-K (Lee et al., 2009), generating plasmid pIZ2031 (Table 2). The prefix pIZ was registered at the Plasmid Reference Center in 1986 (Lederberg, 1986). The *lacO* array and the kanamycin resistance cassette were inserted into pSLT using the lambda Red recombination system (Datsenko and Wanner, 2000) and primers finO1 and finO2 (Supplementary Table S1). Plasmid pWX17, obtained also from David Sherratt’s laboratory, is a pUC18 derivative that carries the yellow fluorescent protein (*yfp*) gene under the control of an arabinose-dependent promoter (Wang et al., 2005). Introduction of pWX17 into appropriate strains was achieved by transformation. Induction of the arabinose-dependent promoter was achieved with 0.02% L-arabinose (Sigma-Aldrich, St. Louis, Missouri). Bacterial cultures were grown in Bertani’s lysogeny broth (LB) at 37°C with shaking.

**Table 1:** *Salmonella enterica* strains constructed for this study.

Strain	Relevant traits
SV8214	pSLT 48 × *lacO* (Km^R^)
SV8216	pSLT/pWX17 (*lacI*::YFP)
SV8217	pSLT 48 × *lacO* (Km^R^)/pWX17 (*lacI*::YFP)
SV8398	pSLT 48 × *lacO ΔparA*::Km^R^/pWX17 (*lacI*::YFP)
SV8960	pSLT-GFP (constitutive *gfp* gene inserted at the *traA* locus; Km^R^)

**Table 2:** Plasmids used in strain constructions.

Name	Relevant traits	Reference or source
pDOC-K	Kanamycin resistance cassette (Km^R^)	Lee et al., 2009
p48LacO	48 *lac* operator array	Lau et al., 2003
pIZ2031	pDOC-K-48x*lacO* (Km^R^ Ap^R^)	This study
pWX17	*lacI*::YFP (Ap^R^)	Garcia-Quintanilla et al., 2006

### Determination of Plasmid Copy Number by Quantitative PCR

DNA extraction was performed using a method that does not modify the chromosome/plasmid DNA ratio (Brandi et al., 2000). Aliquots of crude cell extracts were diluted and subjected to quantitative PCR to estimate the relative content of chromosomal and plasmid DNA. Approximately, 10 ng total DNA were used for each amplification. Quantitative RT-PCR reactions were performed in a Light Cycler 480 II apparatus (Roche). Real-time cycling conditions were as described elsewhere (Camacho et al., 2005). Primers (Supplementary Table S1) were designed with Primer3Plus software.^1^

### *In vivo* Visualization of pSLT by Fluorescence Microscopy

Strains were grown at 37°C in LB and diluted to 1:100 in fresh medium. Samples of 1.5 ml were collected by centrifugation at 3,400 × *g* for 5 min at different optical densities (OD_600_) along the growth cycle. YFP-LacI was induced for 30 min with 0.02% L-arabinose. Cells were stained with 1 μg/ml FM4-64 and 5 μg/ml Hoechst 33258 in 10 μl of mounting medium (40% glycerol in 0.02 M PBS, pH 7.5) and fixed onto a poly-lysine-treated coverslip. Images were obtained by using an Olympus IX-70 Delta Vision fluorescence microscope equipped with a 100X UPLS Apo Objective. Pictures were taken using a CoolSNAP HQ/ICX285 camera and analyzed using ImageJ software (Wayne Rasband, Research Services Branch, Maryland).

### Flow Cytometry

Strain SV8960 was grown at 37°C in LB. At appropriate times, samples of 1.5 ml were collected by centrifugation, washed, and re-suspended in phosphate-buffered saline (PBS) for flow cytometry analysis. Data acquisition was performed using a Cytomics FC500-MPL cytometer (Beckman Coulter, Brea, California), and data were analyzed with FlowJo X, version 10.0.7r software (Tree Star, Oregon).

### Determination of Bacterial Cell Volume

Micrographs of phase-contrast images were used to measure the length and the width of the cells using ImageJ software. Volumes of bacterial cells were calculated as described elsewhere (Volkmer and Heinemann, 2011). For each condition, the dimensions of 150–300 individual cells were measured.

### Serum Treatment

Overnight cultures grown at 37°C in LB were diluted to 1:100 in fresh medium and incubated at 37°C with 200 rpm shaking for 1 h. Cultures were split into two and mouse serum (Sigma) was added to one of the cultures to reach a final concentration of 8%. Samples of 1.5 ml were collected at different optical densities (OD_600_), and treated as described above for visualization by fluorescence microscopy.

### Statistical Analysis

Averages, medians, SDs, and interquartile ranges in the number of pSLT foci were calculated upon examination of 150–300 cells. The exact numbers of cells are indicated in the figure legends. A robust coefficient of variation (rCV) was obtained by dividing the interquartile range by the median of green fluorescent protein (GFP) fluorescence intensity, a parameter calculated with FlowJo X software (Shapiro, 2003).

## Results

### Determination of Plasmid Copy Number by Quantitative PCR

The copy number of plasmid pSLT was determined using a quantitative PCR protocol. Oligonucleotide primers were designed to amplify a gene located relatively close to the origin of replication of the *S. enterica* chromosome (*arcA*), a gene located near the replication terminus (*hisD*), and the *traJ* and *ccdB* genes of pSLT. The PCR efficiency (E) for each primer was calculated from amplification slopes. They were found to be similar (−0.44 < −log E < −0.39), thereby allowing the comparison of results. Threshold cycles were normalized, indicating the relative copy number of pSLT per chromosome. DNA samples were obtained at different growth stages in LB broth, and amplifications were performed in triplicate.

Amplification of pairwise combinations of chromosomal and plasmid-borne genes provided an estimate of the relative number of pSLT copies per chromosome. Such numbers ranged from 0.92 to 1.67 pSLT copies per chromosome (Table 3), with an average of 1.35 plasmid copies per chromosome equivalent. The SDs obtained with different gene pairs and at different growth stages were small, suggesting that the average number of pSLT copies per chromosome remained fairly constant throughout growth. However, this analysis left open the possibility that cells with higher numbers of pSLT copies were overlooked, especially if such cells were rare.

**Table 3:** Number of plasmid of *Salmonella enterica* serovar Typhimurium (pSLT) copies per chromosome, determined by qPCR.

Time	traJ/arcA^a^	traJ/hisD^b^	ccdB/arcA^a^	ccdB/hisD^b^
0	1.12 ± 0.23	1.27 ± 0.32	1.21 ± 0.12	1.39 ± 0.08
60	0.92 ± 0.19	1.06 ± 0.24	1.03 ± 0.31	1.16 ± 0.12
120	1.03 ± 0.28	1.55 ± 0.18	1.17 ± 0.34	1.48 ± 0.32
240	1.29 ± 0.11	1.20 ± 0.35	1.10 ± 0.26	1.67 ± 0.29

a Averages and SDs from five independent experiments

b Averages and SDs from three independent experiments.

### Visualization of pSLT in Live Cells

**Figure 1:**
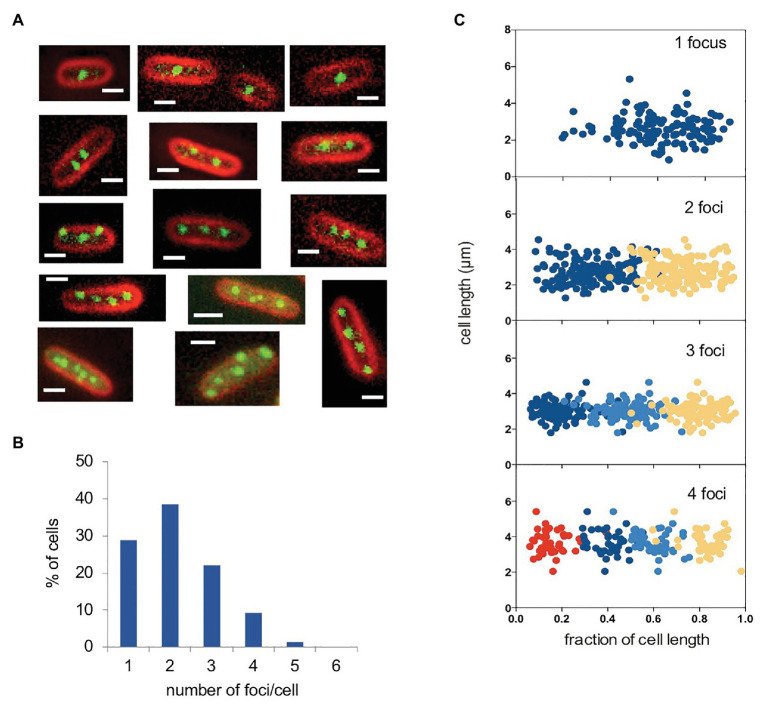
Visualization of the pSLT plasmid tagged with YFP-LacO. **(A)** Fluorescence micrographs of selected cells containing pSLT-LacO plasmids. Cell membranes were stained with FM4-64 (red). YFP-tagged plasmids are visualized as green foci. **(B)** Graphs show cells containing one (28.8%), two (38.5%), three (22.0%), four (9.2%), and five (1.3%). pSLT-LacO fluorescence foci per cell. The number of cells analyzed was 550. **(C)** Subcellular distribution of YFP-tagged pSLT-LacO as a function of cell length in cells containing 1–4 foci. A total of 444 cells were analyzed. Scale bars indicate 1 μm.

Labeling of plasmid pSLT was performed with the Fluorescent Repressor Operator System (FROS; Robinett et al., 1996; Lau et al., 2003), and *in vivo* visualization was achieved by fluorescence microscopy. For this purpose, strain SV8217 was grown overnight at 37°C in LB, diluted to 1:100 in fresh medium, and grown to an OD_600_ of 0.1–0.2. Under these conditions, fluorescence foci formed by the virulence plasmid were visualized (Figure 1A). The majority of cells had either one focus (28%) or two (38%). About 22% of cells had three foci and 4% had four foci (Figure 1B). Cells containing five or more foci were detected at lower frequencies (Figure 1B). As each focus must correspond to one or more plasmid copies, the number of foci can be expected to indicate the minimal number of pSLT copies per cell.

We also examined the subcellular localization of pSLT in cells containing different numbers of foci per cell. In cells containing one focus, it was located at or near mid-cell. In cells containing two foci, they were located at quarter-cell positions. In cells containing three foci, one was located near mid-cell and the other two were located at or near ¼ and ¾ positions. When cells contained four foci, these were located at 1/8, 3/8, 5/8, and 7/8 subcellular positions. A visual summary of these observations is shown in Figure 1C, plotting the position of each focus against the fraction of cell length. We can thus conclude that pSLT is not randomly positioned in the cells, and that its localization pattern depends on the number of foci per cell. Similar localization patterns have been observed in other low-copy-number plasmids (Gordon et al., 1997; Niki and Hiraga, 1997).

**Figure 2:**
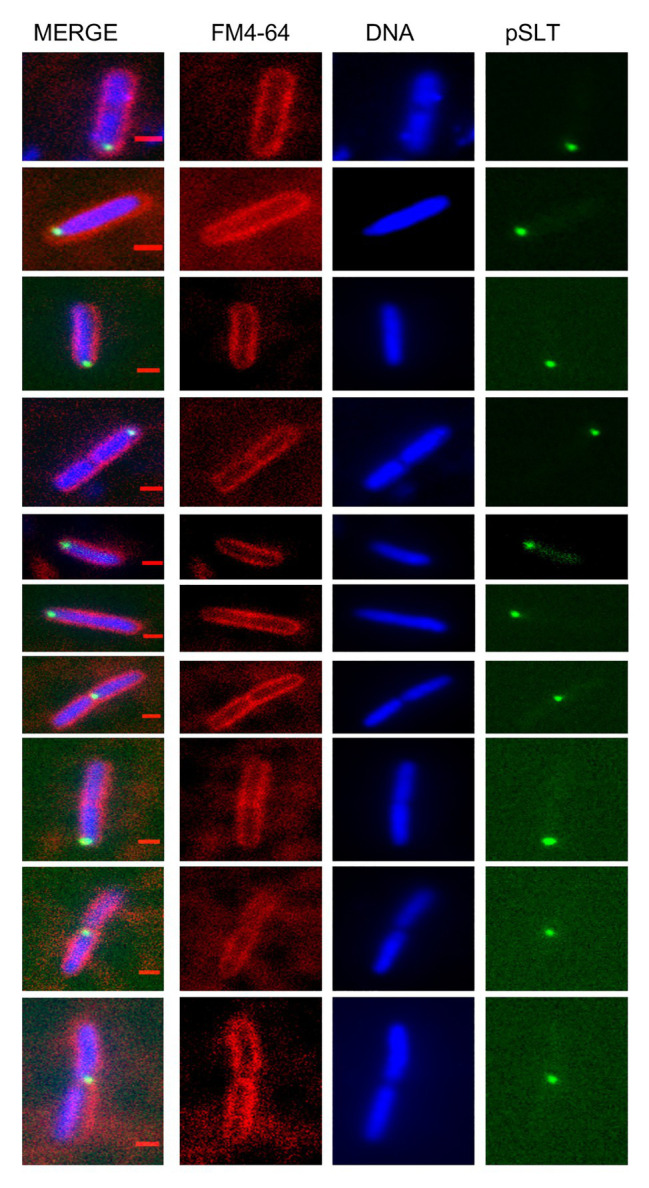
Subcellular localization of the *Salmonella* virulence plasmid in a strain with a defective pSLT partitioning system. Cell membranes were stained with FM4-64 (red), DNA was stained with Hoechst 33258 (blue) and green foci revealed YFP-tagged pSLT. Scale bars indicate 1 μm.

To test whether the subcellular position of pSLT was determined by its partition machinery, we disrupted the *parA* gene, which is known to encode partition functions (Tinge and Curtiss, 1990; Cerin and Hackett, 1993). The pSLT derivative lacking the *parA* gene was detected in spaces not occupied by nucleoids (e.g., near one cell pole; Figure 2). A pattern of this kind is typical upon Par system inactivation in low-copy-number plasmids (Niki and Hiraga, 1997).

### Heterogeneity of pSLT Copy Number Along the Growth Cycle

**Figure 3:**
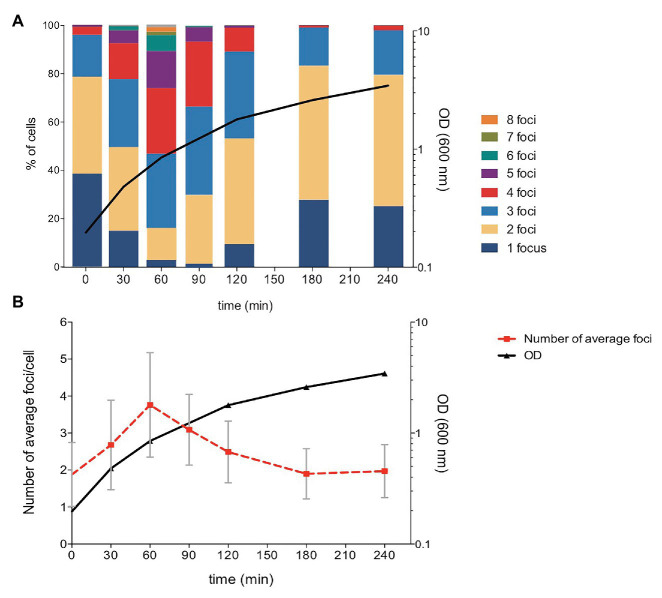
Analysis of the number of pSLT-LacO fluorescent foci during *Salmonella* growth. **(A)** Number of pSLT foci per cell along cell growth. The proportion of cells containing different numbers of foci per cell is shown with a color code. A total of 247, 381, 272, 302, 325, 234, and 206 cells were measured at times 0, 30, 60, 90, 120, 180, and 240 min, respectively. **(B)** Averages and SDs in the number pSLT foci per cell during active growth. Optical density at 600 nm is represented at the right axis in logarithmic scale.

The number of plasmid foci per cell was monitored at different stages of the growth cycle. For this purpose, an overnight culture of strain SV8217 was diluted to 1:100 in fresh LB and grown at 37°C. Samples were periodically taken for cell visualization by fluorescence microscopy. The proportion of cells containing different numbers of fluorescent foci was found to vary along the growth curve, and up to eight foci per cell were found in actively dividing cultures (Figure 3A; Supplementary Figure S1). In contrast, the proportion of cells containing only one focus at mid-exponential phase was only 5% (Figure 3A). Average foci number are shown in Figure 3B, together with SDs that provide quantitative assessment of cell-to-cell heterogeneity in the number of foci.

The number of pSLT foci was also monitored in the presence of mouse serum, a laboratory condition that mimics conditions encountered by *S. enterica* inside animals (Murray et al., 2005). Choice of serum to monitor virulence-related conditions was also based upon the fact that two pSLT genes, *rsk* and *rck*, contribute to serum resistance (Vandenbosch et al., 1989; Heffernan et al., 1992). Cell-to-cell variation in the number of pSLT foci was detected, with numbers ranging from 1 to 5 foci during early and late exponential growth, and from 1 to 8 foci in mid exponential phase (Supplementary Table S2). These reductionist trials provide evidence that cell-to-cell variation in pSLT copy number may occur during infection.

**Figure 4:**
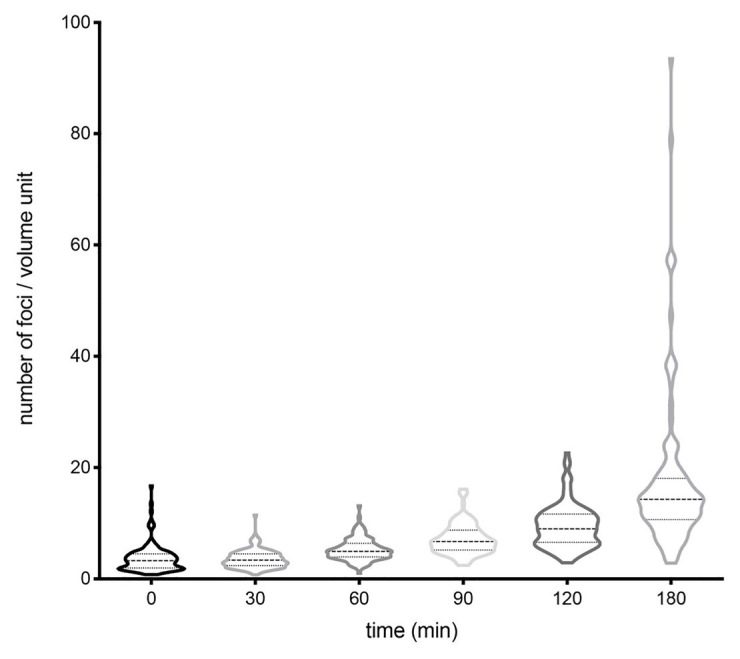
Evolution of the number of foci per cell volume unit along the growth cycle, represented using violin plots. The number of cells analyzed at each time was 150–300. Medians and interquartile ranges are shown. The proportions of cells that fell in tails at 0, 30, 60, 90, 120, and 180 were 21.26, 22.56, 17.65, 16.10, 22.02, and 20.39%, respectively.

To investigate a potential correlation between the number of plasmid foci and the bacterial cell volume, we measured the volume of *S. enterica* cells containing different numbers of foci. Volume measurements were done at different growth stages, and the results are shown in Figure 4 and Supplementary Figure S2. A clear-cut correlation was found between the average number of foci and the volume of the bacterial cell. In contrast, a correlation between the degree of heterogeneity in the number of foci and the cell volume was not detected, suggesting that plasmid copy number heterogeneity may be independent of the cell volume.

### Effect of pSLT Copy Number Variation on Gene Expression Heterogeneity

**Figure 5:**
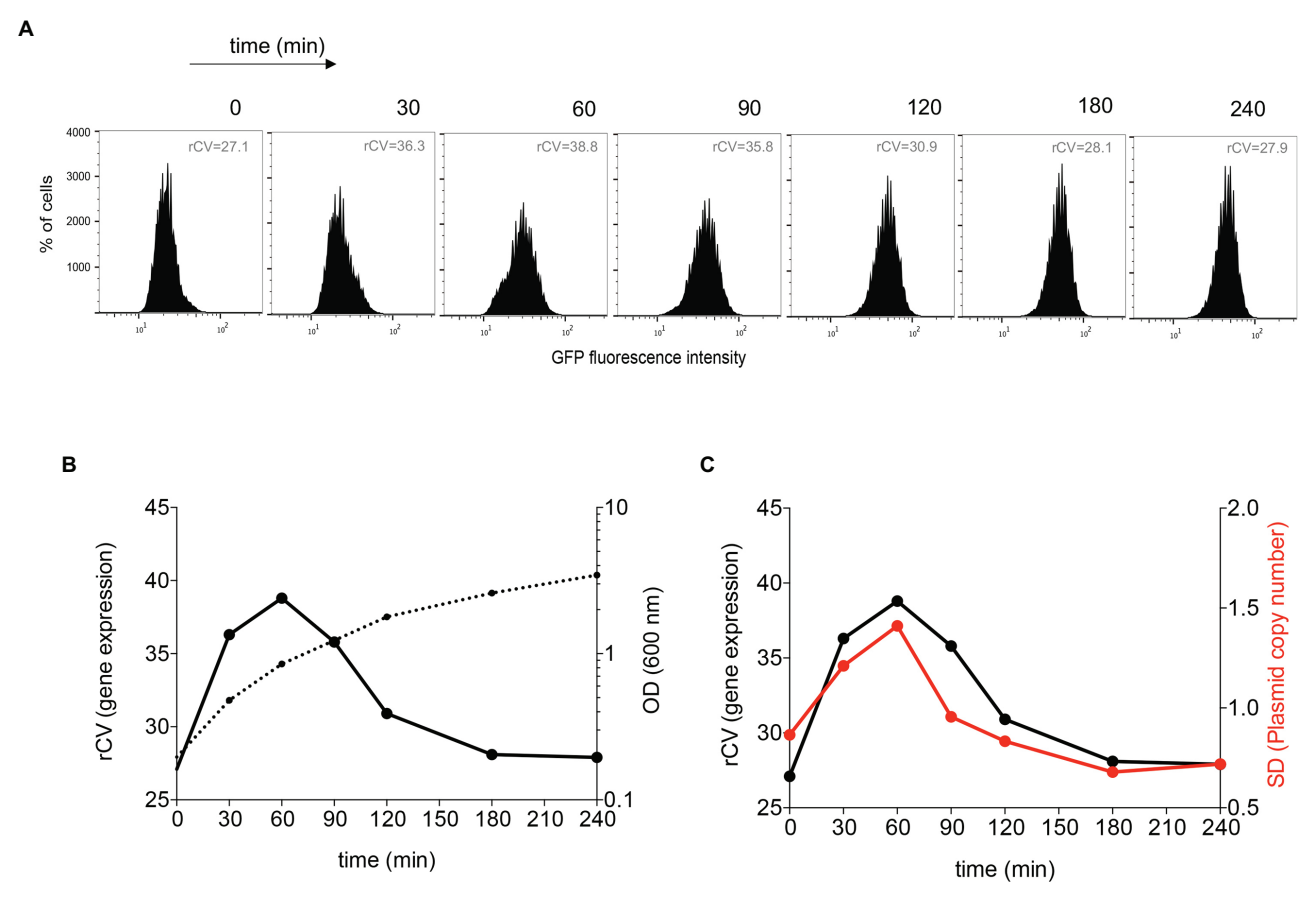
Flow cytometry analysis of pSLT-GFP expression during growth. **(A)** GFP fluorescence intensity distribution in strain SV8960 (pSLT-GFP) grown in LB at 37°C. Robust coefficients of variation (rCVs) are indicated inside graphs. **(B)** Analysis of the degree of heterogeneity in GFP expression along the growth cycle. The left axis displays rCVs of GFP fluorescence histograms, and the right axis represents the optical density at 600 nm. **(C)** Comparative analysis of the degree of heterogeneity in GFP expression and the level of variability in the number of plasmid foci along the growth cycle. The left axis represents the robust coefficient of variation (rCV) of GFP fluorescence histograms, and the right axis represents the SD of the number of pSLT-LacO fluorescence foci per cell.

Differences in gene dosage caused by copy number heterogeneity can be expected to produce cell-to-cell differences in the expression of plasmid-borne genes. To test this prediction, we cloned a constitutive green fluorescent protein (*gfp*) gene on pSLT and monitored cell-to-cell variation in the GFP level using flow cytometry. Cell-to-cell variations in GFP fluorescence were higher upon active growth (Figures 5A,B). To obtain a quantitative estimation of the noise generated by copy number variation, rCVs were calculated. Heterogeneity in the number of foci during active growth correlates with higher noise (Figure 5C), and the higher the variation in plasmid copy number, the higher the coefficient of variation in GFP expression. This study thus suggests that pSLT-borne genes can be a source of gene expression noise due to variation in the plasmid copy number.

## Discussion

Quantitative PCR amplification of plasmid-borne and chromosomal DNA sequences reveals that the virulence plasmid of *Salmonella enterica* serovar Typhimurium, pSLT, has ~1 copy per chromosome like other F-like plasmids (Table 3). However, PCR-based calculation provides a copy number average and does not detect copy number variations among bacterial cells, especially if such cells are rare. In this study, we have exploited the FROS method (Robinett et al., 1996; Lau et al., 2003) to label the pSLT virulence plasmid with the aim of visualizing pSLT foci in individual *Salmonella* cells. The number of fluorescent foci indicates the minimal number of pSLT copies as partially replicated plasmids cannot be detected, and formation of plasmid aggregates is also possible.

Despite the possession of active partition systems that promote faithful distribution of one or few pSLT copies to daughter cells (Tinge and Curtiss, 1990; Lobato-Marquez et al., 2015), remarkable levels of copy number heterogeneity were detected both in LB and in the presence of mouse serum, especially in actively dividing cultures (Figures 1, 3; Supplementary Table S2). Hence, copy number heterogeneity does not seem to depend on specific growth conditions.

A correlation was found between the number of foci and the cell volume, in agreement with the classical view that plasmid replication control responds to cell volume (Chattoraj, 2000; Paulsson, 2002). In contrast, the degree of heterogeneity in the number of foci was found to be independent of the cell volume, suggesting that copy number heterogeneity may have stochastic origin. In support of this view, an analogy can be drawn between stochastic variation in the copy number of low-copy-number plasmids and other cellular processes involving low numbers of molecules (Elowitz et al., 2002; Raser and O’Shea, 2005).

As a consequence of pSLT copy number heterogeneity, the expression of a plasmid-borne *gfp* gene showed large differences from cell to cell (Figure 5). This observation suggests that noisy gene expression of plasmid-borne genes may occur as a consequence of copy number heterogeneity. Due to a finite number effect, low-copy-number plasmids with a partitioning system may actually produce more cellular noise than random partitioning of high-copy-number plasmids (Wong et al., 2010).

At this stage, we do not know whether variation in pSLT copy number occurs also in natural environments. If that is the case, pSLT copy number heterogeneity could be expected to be frequent in animal niches in which *Salmonella* replicates actively such as the small intestine (Sanchez-Romero and Casadesus, 2018) and the gall bladder (Urdaneta et al., 2019). Potential advantages of phenotypic heterogeneity and concomitant noisy gene expression during *Salmonella* infection are thus conceivable. For instance, formation of *Salmonella* cells with increased dosage of conjugation proteins might contribute to the high rates of pSLT transfer detected in the murine ileum (Garcia-Quintanilla et al., 2008). High production of invasion factor Rck by a subpopulation of cells might in turn permit division of labor in a manner analogous to bistable expression of *Salmonella* pathogenicity island 1 (Diard et al., 2013). It is likewise conceivable that production of Pef fimbriae by a subset of cells might produce a bacterial subpopulation able to colonize specific eukaryotic surfaces, a strategy described for fimbrial systems with heterogeneous expression (Suwandi et al., 2019). Whatever the case, speculations on the adaptive value of copy number variation may be justified by the evidence that phenotypic heterogeneity is often an adaptive trait in bacterial populations (Veening et al., 2008; Casadesus and Low, 2013; Weigel and Dersch, 2018).

## Data Availability Statement

The raw data supporting the conclusions of this article will be made available by the authors, without undue reservation.

## Author Contributions

MS-R and ÁM-F carried out the experiments. MS-R, ÁM-F, and JC designed the experiments, interpreted results, and wrote the manuscript. All authors contributed to the article and approved the submitted version.

### Conflict of Interest

The authors declare that the research was conducted in the absence of any commercial or financial relationships that could be construed as a potential conflict of interest.

## Footnotes

^1^http://www.bioinformatics.nl/cgi-bin/primer3plus/primer3plus.cgi/

## References

[ref1] Baumler A. J. Tsolis R. M. Bowe F. A. Kusters J. G. Hoffmann S. Heffron F. (1996). The *pef* fimbrial operon of *Salmonella typhimurium* mediates adhesion to murine small intestine and is necessary for fluid accumulation in the infant mouse. Infect. Immun. 64, 61–68. 10.1128/IAI.64.1.61-68.1996, PMID: 8557375 PMC173728

[ref2] Baumler A. J. Tsolis R. M. Ficht T. A. Adams L. G. (1998). Evolution of host adaptation in *Salmonella enterica*. Infect. Immun. 66, 4579–4587. 10.1128/IAI.66.10.4579-4587.1998, PMID: 9746553 PMC108564

[ref3] Brandi L. Falconi M. Ripa S. (2000). Plasmid curing effect of trovafloxacin. FEMS Microbiol. Lett. 184, 297–302. 10.1111/j.1574-6968.2000.tb09030.x, PMID: 10713437

[ref4] Camacho E. M. Serna A. Madrid C. Marques S. Fernandez R. de la Cruz F.. (2005). Regulation of *finP* transcription by DNA adenine methylation in the virulence plasmid of *Salmonella enterica*. J. Bacteriol. 187, 5691–5699. 10.1128/JB.187.16.5691-5699.2005, PMID: 16077115 PMC1196074

[ref5] Canals R. Chaudhuri R. R. Steiner R. E. Owen S. V. Quinones-Olvera N. Gordon M. A.. (2019). The fitness landscape of the African *Salmonella* Typhimurium ST313 strain D23580 reveals unique properties of the pBT1 plasmid. PLoS Pathog. 15:e1007948. 10.1371/journal.ppat.1007948, PMID: 31560731 PMC6785131

[ref6] Casadesus J. Low D. A. (2013). Programmed heterogeneity: epigenetic mechanisms in bacteria. J. Biol. Chem. 288, 13929–13935. 10.1074/jbc.R113.472274, PMID: 23592777 PMC3656251

[ref7] Cerin H. Hackett J. (1993). The *parVP* region of the *Salmonella typhimurium* virulence plasmid pSLT contains four loci required for incompatibility and partition. Plasmid 30, 30–38. 10.1006/plas.1993.1031, PMID: 8378444

[ref8] Chattoraj D. K. (2000). Control of plasmid DNA replication by iterons: no longer paradoxical. Mol. Microbiol. 37, 467–476. 10.1046/j.1365-2958.2000.01986.x, PMID: 10931340

[ref9] Cirillo D. M. Heffernan E. J. Wu L. Harwood J. Fierer J. Guiney D. G. (1996). Identification of a domain in Rck, a product of the *Salmonella typhimurium* virulence plasmid, required for both serum resistance and cell invasion. Infect. Immun. 64, 2019–2023. 10.1128/IAI.64.6.2019-2023.1996, PMID: 8675302 PMC174031

[ref10] Datsenko K. A. Wanner B. L. (2000). One-step inactivation of chromosomal genes in *Escherichia coli* K-12 using PCR products. Proc. Natl. Acad. Sci. U. S. A. 97, 6640–6645. 10.1073/pnas.120163297, PMID: 10829079 PMC18686

[ref11] Diard M. Garcia V. Maier L. Remus-Emsermann M. N. Regoes R. R. Ackermann M.. (2013). Stabilization of cooperative virulence by the expression of an avirulent phenotype. Nature 494, 353–356. 10.1038/nature11913, PMID: 23426324

[ref12] Elowitz M. B. Levine A. J. Siggia E. D. Swain P. S. (2002). Stochastic gene expression in a single cell. Science 297, 1183–1186. 10.1126/science.1070919, PMID: 12183631

[ref13] Emond-Rheault J. G. Hamel J. Jeukens J. Freschi L. Kukavica-Ibrulj I. Boyle B.. (2020). The *Salmonella enterica* plasmidome as a reservoir of antibiotic resistance. Microorganisms 8:1016. 10.3390/microorganisms8071016, PMID: 32650601 PMC7409225

[ref14] Garcia-Quintanilla M. Casadesus J. (2011). Virulence plasmid interchange between strains ATCC 14028, LT2, and SL1344 of *Salmonella enterica* serovar Typhimurium. Plasmid 65, 169–175. 10.1016/j.plasmid.2010.12.001, PMID: 21145349

[ref15] Garcia-Quintanilla M. Prieto A. I. Barnes L. Ramos-Morales F. Casadesus J. (2006). Bile-induced curing of the virulence plasmid in *Salmonella enterica* serovar Typhimurium. J. Bacteriol. 188, 7963–7965. 10.1128/JB.00995-06, PMID: 16963576 PMC1636308

[ref16] Garcia-Quintanilla M. Ramos-Morales F. Casadesus J. (2008). Conjugal transfer of the *Salmonella enterica* virulence plasmid in the mouse intestine. J. Bacteriol. 190, 1922–1927. 10.1128/JB.01626-07, PMID: 18178735 PMC2258861

[ref17] Garcillan-Barcia M. P. Francia M. V. de la Cruz F. (2009). The diversity of conjugative relaxases and its application in plasmid classification. FEMS Microbiol. Rev. 33, 657–687. 10.1111/j.1574-6976.2009.00168.x, PMID: 19396961

[ref18] Gordon G. S. Sitnikov D. Webb C. D. Teleman A. Straight A. Losick R.. (1997). Chromosome and low copy plasmid segregation in *E. coli*: visual evidence for distinct mechanisms. Cell 90, 1113–1121. 10.1016/S0092-8674(00)80377-3, PMID: 9323139

[ref19] Guerra B. Soto S. Helmuth R. Mendoza M. C. (2002). Characterization of a self-transferable plasmid from *Salmonella enterica* serotype Typhimurium clinical isolates carrying two integron-borne gene cassettes together with virulence and drug resistance genes. Antimicrob. Agents Chemother. 46, 2977–2981. 10.1128/AAC.46.9.2977-2981.2002, PMID: 12183256 PMC127424

[ref20] Guiney D. G. Fang F. C. Krause M. Libby S. (1994). Plasmid-mediated virulence genes in non-typhoid *Salmonella* serovars. FEMS Microbiol. Lett. 124, 1–9. 10.1111/j.1574-6968.1994.tb07253.x, PMID: 8001760

[ref21] Gulig P. A. Danbara H. Guiney D. G. Lax A. J. Norel F. Rhen M. (1993). Molecular analysis of *spv* virulence genes of the *Salmonella* virulence plasmids. Mol. Microbiol. 7, 825–830. 10.1111/j.1365-2958.1993.tb01172.x, PMID: 8483415

[ref22] Heffernan E. J. Reed S. Hackett J. Fierer J. Roudier C. Guiney D. (1992). Mechanism of resistance to complement-mediated killing of bacteria encoded by the *Salmonella typhimurium* virulence plasmid gene rck. J. Clin. Invest. 90, 953–964. 10.1172/JCI115972, PMID: 1522243 PMC329951

[ref23] Huttener M. Prieto A. Aznar S. Bernabeu M. Glaria E. Valledor A. F.. (2019). Expression of a novel class of bacterial Ig-like proteins is required for IncHI plasmid conjugation. PLoS Genet. 15:e1008399. 10.1371/journal.pgen.1008399, PMID: 31527905 PMC6764697

[ref24] Jones G. W. Rabert D. K. Svinarich D. M. Whitfield H. J. (1982). Association of adhesive, invasive, and virulent phenotypes of *Salmonella typhimurium* with autonomous 60-megadalton plasmids. Infect. Immun. 38, 476–486. 10.1128/IAI.38.2.476-486.1982, PMID: 6128304 PMC347764

[ref25] Lau I. F. Filipe S. R. Soballe B. Okstad O. A. Barre F. X. Sherratt D. J. (2003). Spatial and temporal organization of replicating *Escherichia coli* chromosomes. Mol. Microbiol. 49, 731–743. 10.1046/j.1365-2958.2003.03640.x, PMID: 12864855

[ref26] Lederberg E. M. (1986). Plasmid prefix designations registered by the plasmid reference center 1977-1985. Plasmid 15, 57–92. 10.1016/0147-619X(86)90014-4, PMID: 3952192

[ref27] Lee D. J. Bingle L. E. Heurlier K. Pallen M. J. Penn C. W. Busby S. J.. (2009). Gene doctoring: a method for recombineering in laboratory and pathogenic *Escherichia coli* strains. BMC Microbiol. 9:252. 10.1186/1471-2180-9-252, PMID: 20003185 PMC2796669

[ref28] Lian X. Wang X. Liu X. Xia J. Fang L. Sun J.. (2019). oqxAB-positive IncHI2 plasmid pHXY0908 increase *Salmonella enterica* serotype Typhimurium strains tolerance to ciprofloxacin. Front. Cell. Infect. Microbiol. 9:242. 10.3389/fcimb.2019.00242, PMID: 31334135 PMC6617520

[ref29] Lobato-Marquez D. Moreno-Cordoba I. Figueroa V. Diaz-Orejas R. Garcia-del Portillo F. (2015). Distinct type I and type II toxin-antitoxin modules control *Salmonella* lifestyle inside eukaryotic cells. Sci. Rep. 5:9374. 10.1038/srep09374, PMID: 25792384 PMC4366850

[ref30] Mendoza M. Herrero A. Rodicio M. R. (2009). Evolutionary engineering in *Salmonella*: emergence of hybrid virulence-resistance plasmids in non-typhoid serotypes. Enferm. Infecc. Microbiol. Clin. 27, 37–43. 10.1016/j.eimc.2008.09.001, PMID: 19218002

[ref31] Murray G. L. Attridge S. R. Morona R. (2005). Inducible serum resistance in *Salmonella typhimurium* is dependent on *wzz(fepE)*-regulated very long O antigen chains. Microbes Infect. 7, 1296–1304. 10.1016/j.micinf.2005.04.015, PMID: 16027021

[ref32] Niki H. Hiraga S. (1997). Subcellular distribution of actively partitioning F plasmid during the cell division cycle in *E. coli*. Cell 90, 951–957. 10.1016/s0092-8674(00)80359-1, PMID: 9298906

[ref33] Paulsson J. (2002). Multileveled selection on plasmid replication. Genetics 161, 1373–1384. PMID: 12238464 10.1093/genetics/161.4.1373 PMC1462198

[ref34] Pilla G. Tang C. M. (2018). Going around in circles: virulence plasmids in enteric pathogens. Nat. Rev. Microbiol. 16, 484–495. 10.1038/s41579-018-0031-2, PMID: 29855597

[ref35] Raser J. M. O’Shea E. K. (2005). Noise in gene expression: origins, consequences, and control. Science 309, 2010–2013. 10.1126/science.1105891, PMID: 16179466 PMC1360161

[ref36] Robinett C. C. Straight A. Li G. Willhelm C. Sudlow G. Murray A.. (1996). *In vivo* localization of DNA sequences and visualization of large-scale chromatin organization using *lac* operator/repressor recognition. J. Cell Biol. 135, 1685–1700. 10.1083/jcb.135.6.1685, PMID: 8991083 PMC2133976

[ref37] Rotger R. Casadesus J. (1999). The virulence plasmids of *Salmonella*. Int. Microbiol. 2, 177–184. PMID: 10943411

[ref38] Sanchez-Romero M. A. Casadesus J. (2018). Contribution of SPI-1 bistability to *Salmonella enterica* cooperative virulence: insights from single cell analysis. Sci. Rep. 8:14875. 10.1038/s41598-018-33137-z, PMID: 30291285 PMC6173691

[ref39] Shapiro H. M. (2003). Practical flow cytometry. Hoboken, New Jersey: John Wiley & Sons.

[ref40] Suwandi A. Galeev A. Riedel R. Sharma S. Seeger K. Sterzenbach T.. (2019). Std fimbriae-fucose interaction increases *Salmonella*-induced intestinal inflammation and prolongs colonization. PLoS Pathog. 15:e1007915. 10.1371/journal.ppat.1007915, PMID: 31329635 PMC6675130

[ref41] Tinge S. A. Curtiss R. 3rd. (1990). Conservation of *Salmonella typhimurium* virulence plasmid maintenance regions among *Salmonella* serovars as a basis for plasmid curing. Infect. Immun. 58, 3084–3092. 10.1128/IAI.58.9.3084-3092.1990, PMID: 2167294 PMC313615

[ref42] Urdaneta V. Hernandez S. B. Casadesus J. (2019). Mutational and non mutational adaptation of *Salmonella enterica* to the gall bladder. Sci. Rep. 9:5203. 10.1038/s41598-019-41600-8, PMID: 30914708 PMC6435676

[ref43] Vandenbosch J. L. Rabert D. K. Kurlandsky D. R. Jones G. W. (1989). Sequence analysis of *rsk*, a portion of the 95-kilobase plasmid of *Salmonella typhimurium* associated with resistance to the bactericidal activity of serum. Infect. Immun. 57, 850–857. 10.1128/IAI.57.3.850-857.1989, PMID: 2645213 PMC313188

[ref44] Veening J. W. Smits W. K. Kuipers O. P. (2008). Bistability, epigenetics, and bet-hedging in bacteria. Annu. Rev. Microbiol. 62, 193–210. 10.1146/annurev.micro.62.081307.163002, PMID: 18537474

[ref45] Volkmer B. Heinemann M. (2011). Condition-dependent cell volume and concentration of *Escherichia coli* to facilitate data conversion for systems biology modeling. PLoS One 6:e23126. 10.1371/journal.pone.0023126, PMID: 21829590 PMC3146540

[ref46] Wang X. Possoz C. Sherratt D. J. (2005). Dancing around the divisome: asymmetric chromosome segregation in *Escherichia coli*. Genes Dev. 19, 2367–2377. 10.1101/gad.345305, PMID: 16204186 PMC1240045

[ref47] Weigel W. A. Dersch P. (2018). Phenotypic heterogeneity: a bacterial virulence strategy. Microbes Infect. 20, 570–577. 10.1016/j.micinf.2018.01.008, PMID: 29409898

[ref48] Wong N. C. Chatenay D. Robert J. Poirier M. G. (2010). Plasmid copy number noise in monoclonal populations of bacteria. Phys. Rev. 81:011909. 10.1103/PhysRevE.81.011909, PMID: 20365401

