# Corrigendum: Copy Number Heterogeneity in the Virulence Plasmid of *Salmonella enterica*

**DOI:** 10.3389/fmicb.2020.645054

**Published:** 2021-01-14

**Authors:** María A. Sánchez-Romero, Ángela Mérida-Floriano, Josep Casadesús

**Affiliations:** Departamento de Genética, Facultad de Biología, Universidad de Sevilla, Seville, Spain

**Keywords:** Salmonella, virulence plasmid, copy number, phenotypic heterogeneity, noise

In the original article, there was a mistake in the heading of Table 3 as published: in the first column on the left, O.D._600_ is incorrect. The correct heading is Time. The corrected Table 3 appears below.

**Table 3:** Number of plasmid of *Salmonella enterica* serovar Typhimurium (pSLT) copies per chromosome, determined by qPCR.

**Time**	**traJ/arcA^**a**^**	**traJ/hisD^**b**^**	**ccdB/arcA^**a**^**	**ccdB/hisD^**b**^**
0	1.12 ± 0.23	1.27 ± 0.32	1.21 ± 0.12	1.39 ± 0.08
60	0.92 ± 0.19	1.06 ± 0.24	1.03 ± 0.31	1.16 ± 0.12
120	1.03 ± 0.28	1.55 ± 0.18	1.17 ± 0.34	1.48 ± 0.32
240	1.29 ± 0.11	1.20 ± 0.35	1.10 ± 0.26	1.67 ± 0.29

a *Averages and standard deviations from 5 independent experiments*.

b *Averages and standard deviations from 3 independent experiments*.

In the original article, there was an error in the **Abstract**. The corrected sentence should read:

In contrast, heterogeneity in the number of foci appears to be independent of the cell volume and may have stochastic origin.

In the **Introduction** section, paragraph 4, the sentence should read:

Plasmid pSLT has a low (≥1) copy number per cell (Camacho et al., 2005), etc.

In the **Serum Treatment section**, the second sentence should read:

Cultures were split into two…

In the **Heterogeneity of pSLT Copy Number Along the Growth Cycle**, the fifth sentence should read:

Average foci numbers are shown…

In the **Effect of pSLT Copy Number Variation on Gene Expression Heterogeneity** section, the fifth sentence should read:

variation in GFP expression.

The authors apologize for these errors and state that this does not change the scientific conclusions of the article in any way. The original article has been updated.

