# Supplementary material for: Copy Number Heterogeneity in the Virulence Plasmid of Salmonella enterica
Source: Front Microbiol. 2020 Dec 4;11:599931. doi: 10.3389/fmicb.2020.599931 (PMC7746676; doi:10.3389/fmicb.2020.599931)
Supplement: Supplementary file 1 [file Data_Sheet_1.pdf]

Copy number heterogeneity in the virulence plasmid of *Salmonella enterica*

María A. Sánchez-Romero, Ángela Mérida-Floriano
and Josep Casadesús

SUPPLEMENTARY MATERIAL

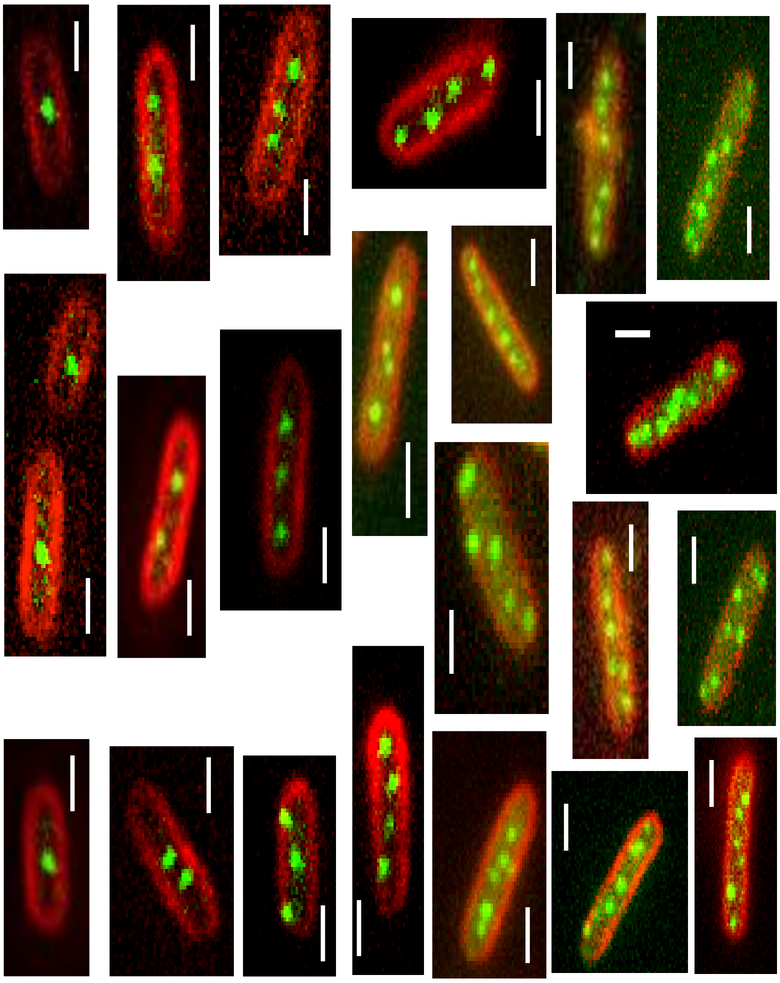

Figure S1. Fluorescence micrographs of *S. enterica* cells containing 1-8 pSLT-LacO foci.

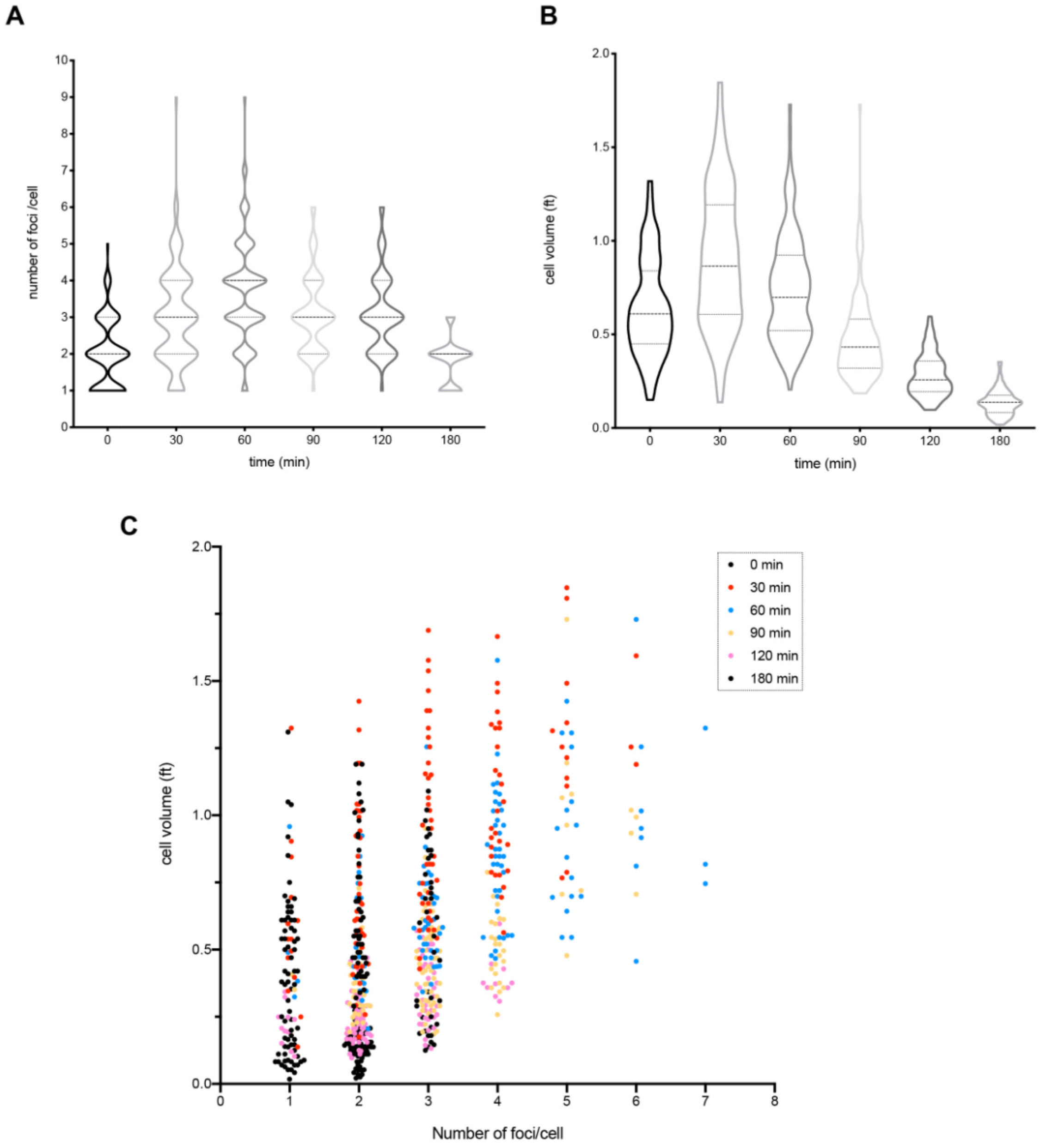

Figure S2. Analysis of cell volume and number of pSLT foci per cell along *Salmonella* growth. The number of cells analyzed at each time was 150-300. **A**. Number of pSLT-LacO fluorescent foci per cell along growth. **B**. Distribution of *S. enterica* cell volumes (ft, μm^3) along growth. **C**. Volumes of cells containing 1-7 pSLT foci at different growth times. Cells with 8 foci are not included because of their low frequency.

Table S1. Oligonucleotides used in this study.

Primer	Sequence (5'-3')
arcA1	CGC AAG CTG AGA TAA ACA GC
arcA2	GTC ATG TT CGC CGA TCA TG
ccdB1	TGA GGT GGC CAG CTT TAT AG
ccdB2	CAG AAA CTC CGC ACA CAG CC
finO1	GCC GTA TTT TGT ACA TCG TTA ACT AAC TAA TTT ACG TCT GGT AAC CAT GAT TAC GCC AAG CTC
finO2	5'GCC GGC GAC CAA GAG GAA TTT CGT AAA TAA CTA TGA GGT AAC AAC GCT AGC ATA TGA GCT CGA
hisD1	CGG TTC CCA GGG ATC CTG CTC ACG CCT G
hisD2	TTA GCG GAT TTC CAG AAA CGG ATG ACC GTT
lacO1	GCC GAA TTC GTA AAA CGA CGG CCA GTG CC
lacO2	CAG GAA ACA GCT ATG ACC ATG GTA CCG
traJ1	CTA AAT CAG GAT AGA TCT ATT ATT CTT C
traJ2	GAA GAA TAA TAG ATC TAT CCT GAT TT AG

Table S2. Percentages of cells showing different numbers of pSLT foci along the cell cycle in the presence and in the absence of mouse serum [a]

	Time (min)													
Number of foci	0		30		60		90		120		180		240	
	LB	Serum	LB	Serum	LB	Serum	LB	Serum	LB	Serum	LB	Serum	LB	Serum
1	38.4	24.8	14.9	16.7	2.9	4.7	1.6	2.5	9.5	12.8	27.7	30.0	25.2	17.5
2	40.0	43.6	34.6	39.4	13.2	24.9	28.4	36.1	43.6	48.6	55.5	57.5	54.3	61.8
3	17.4	25.5	28.0	26.8	30.8	32.4	36.4	37.4	36	27.9	15.8	11.7	18.4	19.8
4	3.2	4.7	14.9	12.2	27.2	18.5	26.8	17.8	9.8	8.2	0.8	0.6	1.9	0.7
5	0.8	1.3	5.4	3.3	15.7	13.4	5.9	4.6	0.9	2.2	0	0	0	0
6	0	0	1.8	0.9	6.6	4.7	0.6	0.8	0	0	0	0	0	0
7	0	0	0	0.3	1.4	0.7	0	0.4	0	0	0	0	0	0
8	0	0	0	0	1.8	0.4	0	0	0	0	0	0	0	0

[a] Numbers of cells examined: >300 in every condition.
